# Person-centered workplace culture: insights from an inpatient department for older adults with chronic illnesses

**DOI:** 10.3389/fmed.2025.1532419

**Published:** 2025-02-26

**Authors:** Diana Vareta, Célia Oliveira, Filipa Ventura

**Affiliations:** ^1^PhD Program, University of Lisbon (UL) and Nursing School of Lisbon (ESEL), Lisbon, Portugal; ^2^Egas Moniz Interdisciplinary Research Centre (CiiEM), Egas Moniz Universitary Institute, Almada, Portugal; ^3^Nursing School of Lisbon (ESEL), Lisbon, Portugal; ^4^The Health Sciences Research Unit: Nursing (UICISA:E), Nursing School of Coimbra (ESEnfC), Coimbra, Portugal

**Keywords:** patient-centered care, workplace cultural critical assessment tool, workplace culture, aged, inpatient, non-communicable diseases

## Abstract

**Introduction:**

An aging population and the increasing prevalence of chronic conditions challenge healthcare systems in developed countries. In response, there is a growing emphasis on person-centered care, as advocated by the World Health Organization and integrated into national health strategies in countries such as the UK and Sweden. However, transitioning to person-centered care is a complex, long-term process shaped by organizational culture and care environments. These contextual factors play crucial roles in the development and sustainability of person-centered practice, significantly transforming the experiences of both older adults and staff.

**Objective:**

To describe how workplace culture within an inpatient hospital department shapes person-centered care practices for older adults with chronic illnesses.

**Methods:**

A qualitative, descriptive, exploratory-observational study was performed. Data were collected through participant observation guided by the Workplace Culture Critical Analysis Tool^®^. In a deductive thematic content analysis, data patterns of meaning were identified. The themes were generated underpinned by the Person-Centered Practice Framework dimensions of prerequisites, the practice environment, and person-centered processes and their respective constructs.

**Results:**

Themes related to all person-centered practice dimensions were identified. Task demands during shifts create tension between routine-oriented work and the holistic, individualized approach required for person-centeredness. The absence of systematic multiprofessional team meetings further exacerbates this issue, limiting collaborative decision-making and personalized care planning. The contrasts in some subthemes may be related to discrepancies in the care provided by different professionals.

**Conclusion:**

This study highlights the tension between routine-driven care and individualized approaches. Addressing identified challenges, such as formalizing multiprofessional meetings and enhancing reflective practices, is crucial for advancing person-centered care in this setting.

## 1 Introduction

The landscape of healthcare services in developed nations is undergoing significant transformation. The rise in chronic conditions among aging populations presents significant challenges to healthcare systems, particularly in ensuring accessible, equitable, and collaborative care (1). In response, Western health systems are increasingly transitioning from biomedical care models to person-centered clinical practices. A shift aimed at addressing these complex, evolving needs.

The World Health Organization (2) underscored the need to adopt person-centered care paradigms, which incorporate the perspectives of individuals, families, and communities. Older adults should be viewed as active participants in shaping services according to their expectations, preferences, and needs, compassionately and comprehensively (2, 3).

Following the lead of certain European nations such as the United Kingdom (4) and Sweden (5), Portugal’s National Health Service acknowledges the imperative of addressing citizens’ needs and expectations, recognizing their involvement in health management processes through initiatives such as the “SNS + Proximity” program (6). The program prioritizes a whole-system approach that puts older adults at the center where healthcare professionals and policymakers must be aligned with this vision. Despite the impetus for change, it is essential to view the transformation of health services toward person-centered practice (PCP) as a long-term process (7).

The Person-Centered Practice Framework (PCPF) (3) is a valuable guide for its implementation and development (8, 9). However, while the concept is understood theoretically, recognizing and embodying it in practice can be challenging (10, 11). Contextual factors such as organizational culture, the learning environment, and the care environment itself pose the most significant demands to the development of cultures that can sustain person-centeredness care due to its potential to restrict or support this practice (12–14).

Workplace culture in healthcare settings is not solely about individuals; rather, it revolves around the social contexts shaping behavior and the accepted norms. Behavior patterns mirror cultural aspects, highlighting the values, beliefs, and assumptions staff embrace. This culture impacts staff and user experiences, staff motivation and effectiveness, evidence implementation in practice, patient safety, innovation adoption, and productivity (15).

This study is part of a clinical study protocol (16) designed to provide recommendations for improving PCP in the daily care of hospitalized older adults with chronic illness at an internal medicine department. It corresponds to the qualitative string, which aims to characterize the workplace culture of an inpatient hospital department with a high prevalence of older adults with chronic illnesses. This study aims to describe how the workplace culture within an inpatient hospital department shapes person-centered care practices for older adults with chronic illnesses. It explores cultural factors influencing daily care experiences for older adults and staff, focusing on contextual challenges and adaptations in chronic care and considering all principles and domains presented in the PCPF.

## 2 Materials and methods

### 2.1 Design, population and study site

A qualitative, descriptive, exploratory-observational approach was followed. The design was informed by the ethnographic tradition, as it allows the investigation of social interactions and sheds light on the contextual factors influencing those interactions and activities. It offers a valuable approach to examining how the environment shapes the daily experiences of a specific population within a given social framework and cultural context. Ethnography, in its alignment with the PCP, delves into the influence of the environment on both older adults and staff, allowing the integration of diverse voices and experiences into a co-constructed research product (17).

The study was conducted at an internal medicine inpatient unit of a secondary hospital in an urban area of Portugal. The internal medicine department provides care for older adults with medical conditions, typically multiple chronic diseases. Accidental sampling methods were used to recruit professionals from the healthcare team working at the unit and older adults hospitalized who fulfilled the defined inclusion criteria during the data collection period.

The internal medicine unit comprises 46 inpatient beds. The physical area comprises nine patient rooms and offices for nurses and physicians along a lengthy corridor. A level 2 medical care unit with a distinct healthcare team is at the corridor end. It also has two single rooms designated for older adults with communicable diseases that meet isolation criteria. The other rooms accommodate three beds each, with a shared bathroom available. There is also a dining hall, the supplies room, and the dressing rooms. The workload distribution of nurses and health assistants prioritizes the level of dependency of the assigned older adults regarding the continuity of care by professionals. The medical staff consists of four different teams, and each team is designated a doctor who follows the patient throughout their hospitalization.

Regarding leadership, the clinical director, who has held the position for 6 years, oversees the coordination of medical teams, clinical decision-making, and overall unit organization. The head nurse, responsible for coordinating the nursing and health assistants’ teams, had been informally managing the role for a year and was officially appointed a month before data collection began.

The team meeting occurs once a week and involves the clinical director, medical team members, the nurse manager, and one nurse specialist providing management support. No registered nurses or physiotherapists are involved. Clinical cases are presented, treatment plans are discussed, and discharges are prepared in these meetings.

Care activities within the ward followed structured routine schedules around three distinct periods: morning, evening, and night. Mornings were oriented to carry out tasks such as care planning, hygiene, treatments, and complementary diagnostic tests, whereas evening, night, and weekend shifts prioritized surveillance and monitoring. Most discharges and admissions take place in the afternoon.

Visiting hours are scheduled from 2 pm to 7 pm during half-hour periods. Visitors are not allowed to stay overnight, except if the patient is disabled or has a terminal illness and if one of the single rooms is available. There is no common space to spend time with family or to talk to other older adults.

The dining hall is used by health professionals but rarely by older adults. The meals are served by a kitchen assistant in each room, who receives the meal plan in advance.

The emergency car is in the corridor and is constantly available.

### 2.2 Inclusion criteria

Older adults were eligible to participate in the study if they were over 65 years old, had a chronic disease diagnosis, were hospitalized at the inpatient internal medicine unit for more than 48 h, and could understand and communicate in Portuguese.

All healthcare professionals working full-time in the internal medicine unit were eligible to participate in the study.

### 2.3 Exclusion criteria

The 6-Item Cognitive Impairment Test (6-CIT) (18) assessed older adults’ cognitive impairment and was the exclusion criterion. The cut-off point considers the level of education, namely a score of up to and including 2 years ≥12, a score of 3 to 6 years ≥10, and a score of more than 7 years ≥4.

### 2.4 Data collection

Data were collected between February and April 2024. A questionnaire including sociodemographic and health history characteristics was provided to the hospitalized older adults, and a questionnaire including sociodemographic and professional characteristics was provided to the healthcare professionals who met the defined criteria. Preparatory visits were made to discuss the study proposal and methodology with staff, familiarize the first researcher with the observation site’s environment, and prepare the record data strategy during observation (notes, audio).

Participant observation was conducted systematically across all weekdays and shift patterns to capture diverse aspects of the department’s culture. Guided by the Workplace Culture Critical Analysis Tool® (WCCAT), observations were performed. The WCCAT is an instrument for systematically generating evidence from observing the interactions between participants and the context, illuminating various aspects of practice (19). It was initially developed underpinned by the Person-Centered Nursing Framework (20) and was reviewed by the International Community of Practice members to ensure its alignment with the current PCPF and further tested for face validity (19). The WCCAT addresses the subjectivity inherent in qualitative data collection, as it combines ethnographic approaches with person-centered principles involving participatory, observational, and reflective elements (21).

For this study, WCCAT was translated into European Portuguese, supported by the translation and cultural adaptation of practice assessment instruments underpinned by the PCPF, such as the Person-centered Practice Inventory-Staff (22) and Person-centered Practice Inventory-Care (23), and back-translated to ensure that the fundamental principles were captured, as recommended by the authors (19). A protocol based on the authors’ guidelines for each phase was previously defined to ensure rigor in using WCCAT (Supplementary material 1).

The observations were planned to include a mix of shorter intervals (5–15 min) for capturing routine daily interactions and longer intervals (up to 60 min) to examine more complex care dynamics. This design aims to ensure a comprehensive view of the department’s cultural environment across different times of day and activity levels.

The first author positioned herself at different locations throughout the corridors, patient rooms, and physicians and nursing offices, strategic for observation while remaining unobtrusive to the situation. She relied on the essential senses: what was seen, heard, smelled or experienced.

After each observation session, an informal debriefing with the healthcare professional engaged in the interaction was conducted to address any issues that arose. This provided staff with an opportunity to discuss the observations and the implications for their practice individually and immediately following the observation period (19).

The saturation of findings was determined when a consistent perception of all the WCCAT domains in the context was obtained, which led to the closure of data collection.

Once the data had been collected, the preliminary results were analyzed, and two reflective sessions with the multiprofessional team were held. These sessions occurred several weeks after the observation phase and were a collaborative effort, where the multiprofessional team reflected on the data obtained and the meaning attributed to it, compared with their perception of the unit’s working culture. The sessions were scheduled to last 1 h, at a time compatible with the unit’s activities, announced 1 week earlier to the different professional groups, and conducted according to the script created for the purpose (Supplementary material 2).

### 2.5 Data analysis

After each observation, descriptions of what was observed were audio-recorded, transcribed, and supplemented with notes and comments. The first researcher begins a comprehensive familiarization of the data, reading it to gain an overall sense and ensure deep understanding. It was then reread, and descriptions and key words were highlighted. The qualitative data analysis software WebQda^®^ (3.0 version, 2016, Aveiro, Portugal), combined with handwriting and mind-mapping methods to assist with developing themes, facilitated the analysis.

Deductive thematic content analysis was performed to identify data patterns of meaning or relations in an interpretive and iterative process data from a preexisting theoretical framework (24, 25). The themes were generated underpinned by the PCPF dimensions of prerequisites, the practice environment, and person-centered processes and their respective constructs. The subthemes arose in line with the data contribution to each theme, aiming to find evidence and examples that align with or challenge these theoretical concepts. During the definition of subthemes, it was ensured that each subtheme resonated with the predefined theoretical concepts (25). The first researcher carried out the data analysis, which was reviewed and refined by all the authors to produce the final set of themes and subthemes used to describe the work culture of the unit, ensuring internal coherence and alignment with the underlying theory.

Regarding the participants’ characteristics, the numerical data was analyzed using the statistical package for social sciences software (IBM SPSS Statistics^®^ for Windows, v.29.0. IBM Corp. Released 2023, Armonk, NY, United States). Normality tests for numeric variables, namely Kolmogorov–Smirnov and Shapiro–Wilk, were conducted to assess data distribution.

### 2.6 Ethical considerations

The study obtained ethical approval from the hospital’s Ethics Committee (ref. nr. 36/2021). All procedures followed the principles outlined in the Declaration of Helsinki (26) and the General Data Protection Regulation (27). Permission to use the WCCAT and 6-CIT was obtained from the respective authors.

Prospective participants received detailed oral and written information about the study, covering its purpose, relevance, data collection methods, expected participation, and data disclosure. Older adults had a minimum 24-h reflection period between receiving information and providing consent. Staff members participated in preparatory visits, and before observation, existing questions were clarified. Their voluntary attendance in reflective sessions implied consent. Participants who declined consent could be present circumstantially in the scene but were not included in the recordings during the observation sessions.

## 3 Results

Approximately 20 h of participant observation, divided into sets of 5 to 60 min (Table 1), were completed across 3 months, covering all weekdays and the two settings that compose the internal medicine unit. The observations occurred during the 24-h shift patterns, predominantly during the morning shift (67%) (Table 1). The most frequently observed situations were care interactions (50%), staff interactions (44%), the dynamics of the unit (33%), and shift handover (11%) (Table 1), with some of them overlapping. Among the participants, nurses were present in most observations (94%), followed by older adults (60%), health assistants (48%), physicians (33%), and physiotherapists (4%) (Table 1).

**Table 1:** Observations characteristics.

Characteristics	%
Time of observation	
Morning shift	67.4
Afternoon shift	30.4
Night shift	2.2
Duration (minutes)	
<15	44.5
[15–30]	30.4
>30	26.1
Participants	
Nurse	93.5
Health assistant	47.8
Physician	32.6
Physiotherapist	4.4
Older adults	60.1
Situation	
Shift handover	10.9
Care interaction	50
Staff interaction	43.5
Unit dynamic	32.6

### 3.1 Characteristics of the participants

A total of 67 participants were observed, including 41 healthcare professionals and 26 older adults. All professional groups were included, with nurses (49%) being the most representative group, followed by physicians (27%), health assistants (22%), and physiotherapists (2%) (Table 2). The participants were predominantly female (81%). Regarding the educational level, 70% of the health professionals are graduates, 10% postgraduate, and 20% undergraduate. In terms of professional experience, 39% of healthcare professionals had less than 5 years of experience, 17% had between 5 and 10 years, 32% had between 10 and 19 years, and 12% had more than 20 years of experience (Table 2).

**Table 2:** Sociodemographic characteristics of the staff.

Characteristics	*N* = 41
Gender	
Female	33 (80.5%)
Male	8 (19.5%)
Profession	
Nurse	20 (48.8%)
Physician	11 (26.8%)
Physiotherapist	1 (2.4%)
Health assistant	9 (22%)
Educational level	
Undergraduate	9 (20%)
Degree	28 (70%)
Postgraduate	4 (10%)
Professional experience (years)	
<5	16 (39%)
[5–9]	7 (17.1%)
[10–19]	13 (31.7%)
[20–40]	5 (12.2%)

All the older adults were Portuguese and were between 65 and 93 years old (*M* = 77.4; SD = 1.5). The sex distribution revealed a predominance of females (65%). Most participants lived primarily in urban areas (62%), in their own homes, or in relatives’ homes (89%). Among the participants living at home, 74% had no domiciliary social or healthcare support, 13% had assistance from healthcare professionals, and 13% received care from informal carers (Table 2). Only 12% of the participants lived in a public or private residential facility that provides nursing care (Table 3).

**Table 3:** Older adults’ sociodemographic characteristics.

Characteristics	*N* = 26
Sex	
Female	17 (65.4%)
Male	9 (34.6%)
Age	
[65, 69]	3 (11.5%)
[70, 74]	7 (26.9%)
[75, 79]	8 (30.8%)
[80, 84]	4 (15.4%)
[85, 89]	0 (0%)
≥90	4 (15.4%)
Living environment	
Rural	10 (38.5%)
Urban	16 (61.5%)
Residence	
Home	23 (88.5%)
Nursing home	3 (11.5%)
Home care	
Health professional	3 (13%)
Informal carer	3 (13%)
None	17 (74%)
Educational level	
Graduate	2 (7.7%)
High school	4 (15.4%)
Middle school	6 (23.1%)
Elementary school	12 (46.1%)
Uneducated	2 (7.7%)

The educational level ranged between no academic education (8%) and graduate (8%), with 46% of older adults with an elementary school education (Table 3). Previous hospitalization experience was referred to by 50% of the participants, who had between 1 and 3 distinct episodes of hospitalization. During the current hospitalization, the median length of hospital stay was 5 days (IQR = 4–7 days) (Table 4).

**Table 4:** Older adults’ health history.

	*N* = 26
Previous hospitalization	
No	13 (50%)
Yes	13 (50%)
Number of previous episodes	
1	5 (35.7%)
2	5 (35.7%)
3	3 (28.6%)
Health history	
Diseases of the circulatory system	15 (57.69%)
Oncological diseases	5 (19.23%)
Diseases of the nervous system	3 (11.54%)
Other diseases	3 (11.54%)
Actual diagnosis	
Diseases of the respiratory system	9 (34.62%)
Metabolic, endocrine, and nutritional diseases	5 (19.23%)
Diseases of the circulatory system	3 (11.53%)
Diseases of the genitourinary system	3 (11.53%)
Diseases of nervous system	3 (11.53%)
Other diseases	3 (11.53%)
Length of stay	
[2, 3]	2 (7.69%)
[4, 6]	16 (61.54%)
[7, 9]	7 (26.92%)
≥10	1 (3.85%)
Barthel index	
Complete independence	3 (11.53%)
Moderate dependence	13 (50.0%)
Severe dependence	9 (34.62%)
Total dependence	1 (3.85%)

The most frequently identified diagnoses among the older adults were respiratory system diseases (35%), followed by metabolic, endocrine, and nutritional diseases (19%). The health history characteristics of the study sample were predominantly related to the circulatory system (58%), followed by oncological diseases (19%).

Regarding the level of dependence during hospitalization, the Barthel index revealed that half of the population had moderate dependence (50%), followed by severe dependence (34.6%), complete independence (11.5%), and total dependence (3.9%) (Table 4).

### 3.2 Reflective sessions

The reflective sessions were attended by 15 participants (9 nurses, 2 physicians and 4 health assistants). During the sessions, the healthcare professionals commented and reflected on their personal experiences, adding information to the data already collected. The results of the reflective sessions were compared with the observational data to validate, complement, or challenge the findings. The collaborative nature of the meetings ensured that healthcare professionals’ perspectives were a key element of the analysis and interpretation process. Globally, the participants supported the findings, considering they are aligned with the working culture experienced in the context.

### 3.3 Thematic analysis findings

In addition to the themes elicited in the PCPF constructs, the thematic analysis uncovered several subthemes that illustrate how the PCP is integrated within the internal medicine unit (Table 5). The findings are organized by themes to clearly present the subthemes within each domain. To enhance understanding, direct speech is italicized, participant roles are indicated (i.e., OA for older adults, HP for health professionals), and any unobservable details are noted within brackets [].

**Table 5:** Themes and subthemes.

Dimension	Theme	Subtheme
Prerequisites	Professionally competent	Diverse depth of field-specific knowledge Expertise in navigating complex situations Blending scientific rigor with informal nuances Competence acknowledged by peers
	Developed interpersonal skills	Effective communication with older adults and families Seamless collaboration through professional communication Mutually supportive interpersonal relationship
	Commitment to the job	Commitment to ensuring quality of care despite disparities Fluctuation in dedication over time Variability in punctuality
	Knowing self	Lack of critical team reflection Difficulty in managing emotional demands
	Clarity of beliefs and values	Influence of personal convictions on professional practice
The practice environment	Appropriate skill mix	Managing patient-to-staff ratio Balancing specialization and experience in care
	Shared decision-making systems	Lack of multiprofessional meetings Opportunities for active involvement in decision-making
	Effective staff relationships	Collaborative teamwork Mutual support among staff
	Power sharing	Equity among professionals within the same group Impaired power sharing between professional groups
	The physical environment	Influence of unit architecture on the care environment Balancing ward dynamics with patient care Challenges related to patient room design
	Supportive organization systems	Promoting staff involvement in practice development Ensuring access to training opportunities Leadership that cultivates collaboration
	Potential for innovation and risk taking	Divergent approaches to healthcare delivery
Person-centered processes	Working with the person’s beliefs and values	Efforts to know older adults Tension between individualized and standardized care
	Sharing decision-making	Exchange of information on care decisions Collaborative decision-making in basic care needs Routine-driven approaches to care transitions
	Engaging authentically	Understanding person’s perspective Aligning care with patient’s known wishes
	Being sympathetically present	Demostrating understanding and support
	Working holistically	Integrating all dimensions of the person in care Recognition of biomedical tasks in practice

The subthemes identified in Prerequisites dimension disclosed different aspects of professional practice, revealing that while healthcare professionals displayed technical competency and effective interpersonal skills, the demands of routines often led to a task-oriented approach that limited holistic care. Professional experience enabled the anticipation of older adults’ needs, yet opportunities for reflective practice and emotional competency were inconsistent. These findings suggest a need for enhanced support for less experienced staff and structured moments for team reflection, fostering both individual growth and the alignment of care with person-centered principles.

#### 3.3.1 Professionally competent

##### 3.3.1.1 Diverse depth of field specific knowledge

The healthcare team evidenced a technical-scientific domain relevant to their respective roles and the ability to share it with others to ensure better quality care.

The lady had a recent fracture of the femoral neck, and the HP put a cushion between her limbs to do the mobilizations and explained to the colleague: “*We leave the cushion so as not to undo the hip angle and one leg is not in contact with the other*.” (Obs. 18)

Nonetheless, the tendency to follow a work routine weakened the importance of this knowledge,

“*The patient was dyspneic, shivering, and feeling unwell; the paracetamol was brought forward from 3 pm due to a suspected temperature rise of 37° (…)*.” The HP receiving the information asked how the temperature was after the medication administration, and the colleague said she had not assessed it as it was time for handover. (Obs. 10)

and a lack of knowledge in specific care areas was identified.

A HP who has heard the persistent NIV alarm comes and says, “*Don’t you need help?*” At the same time, she notices that the halter is on wrong (…) and immediately puts the mask on the woman’s face, repositions the halter (…) and checks that the mask is big enough for the woman’s features. The colleague says, “*I don’t know anything about NIV*.” (Obs. 18)

##### 3.3.1.2 Expertise in navigating complex situations

The team members’ expertise in handling challenging situations, problem-solving, and teamwork was evident among experienced professionals.

The nurse in charge comes to see the patient and immediately positions him in semi-fowler’s [suspecting atelectasis], with improvement in the clinical condition. (Obs. 12)

##### 3.3.1.3 Blending scientific rigor with informal nuances

In most staff interactions, the language used was clear, technical, and scientific.

The HP transmitting the shift information used clear, scientific language with no value judgments. (Obs. 25)

However, colloquial language and some inappropriate expressions were used.

“*was tied up to the neck*” (Obs. 10); “*doesn’t look like a gypsy*” (Obs. 15)

##### 3.3.1.4 Competence acknowledged by peers

Some team members were recognized by peers as references for their ability to facilitate knowledge transfer, skill development, and professional growth among other members.

The younger HP comes to ask for support on the most appropriate treatment for a pressure ulcer. Her colleague says, “*you know who is really good at helping you with that is A.F., who knows a lot about wounds*”. (Obs. 5)

#### 3.3.2 Developed interpersonal skills

##### 3.3.2.1 Effective communication with older adults and families

The staff demonstrated effective communication with older adults and their families, swoed the ability to listen to other perspectives and expressed respect through verbal and non-verbal language.

HP: “*We can call your daughter and (…) ask her to bring some clothes*.” OA: “*Yes, (…) but let her bring some proper clothes*.” HP: “*What do you mean by proper clothes?*” OA: “*My gypsy clothes, with those pretty shirts and the skirt*.” The HP smiles and says, “*Don’t you prefer more comfortable clothes?*” (*…*) The lady says that she prefers to be well-dressed even when hospitalized. The HP agrees, saying, “I will call your daughter away and ask her to bring the clothes.” (Obs. 16)

##### 3.3.2.2 Seamless collaboration through professional communication

Communication between healthcare professionals was characterized by collaboration and effective exchange of information, which fostered a cohesive team dynamic and enhanced inpatient care outcomes. Using their knowledge, the staff generally gave voice to their assessments within the multiprofessional team.

The colleagues listen in silence to the HP who is handing over the shift. There are occasional interruptions to question and validate or obtain more information that they consider pertinent. (Obs. 1)

##### 3.3.2.3 Mutually supportive interpersonal relationship

Healthcare professionals showed open and respectful communication, fostered positive relationships with older adults, and provided reassurance and a sense of security.

OA: “*I am very anxious… and I cannot see myself getting better; I always have a fever*.” The HP says, “*I understand you are feeling anxious, but the bacteria is now identified, and you are taking the right antibiotic. Today, I will ask the doctor to come and talk to you*.” (The HP holds her hand and smiles). (Obs. 2)

#### 3.3.3 Commitment to the job

##### 3.3.3.1 Commitment to ensuring quality of care despite disparities

The desire to improve care quality through evidence-based care and the creation of opportunities to share knowledge between professionals was evident in this context.

When the health assistant leaves the room, the nurse calls her and reinforces the importance of people wearing slippers when walking around the room and toilets. (Obs. 4)

The nurse in charge complements the information provided by the night shift nurse with data from her personal experience (…) and evidence to justify decision-making in relation to the patient (less frequent administration of cleansing enemas due to significant ionic alterations requiring correction with IV potassium). (Obs. 36)

On the other hand, in some situations in which self-will was overridden by compliance with rules regarding staff image and safety.

Wearing artificial nails (Obs. 36), loose hair (Obs. 39), or an open gown (Obs. 39).

##### 3.3.3.2 Fluctuation in dedication over time

The dedication was evident in the professionals’ commitment to spending time with the older adults, getting to know them, and addressing more than just their basic needs.

HP: (…) asks if the patient is comfortable. OA: yes. She seems bored. HP: “*Do you want to turn on the TV? Do you like reading?* She says she has no patience for such things. HP: “*Would you like to talk for a while?*” OA: “*Never mind, you’ve got a lot to do*.” HP: “*Let’s agree on something, I’ll give this lady a bath and when I’ve finished, I’ll come back and talk to you*”. (Obs. 6)

However, not all moments were used to improve relationships with older adults nor to enhance care provision.

When they *determined that the shift’s tasks had been completed*, the HP waited for their colleagues to arrive in the armchair. They stretch their legs and spend time on their cell phones. (Obs. 34)

##### 3.3.3.3 Variability in punctuality

Different behaviors were observed among nursing teams; some arrived in uniform and on time for shifts, while others were late, which interrupted the shift handover and did not allow them to participate from the beginning.

All the staff arrived on time, in uniform, and greeted their colleagues. (Obs. 1)

At 8 a.m., three colleagues arrived in uniform, and the HP started the shift handover. (…) After 5 min, another colleague arrives, also in uniform, and after 15 min, the fifth member arrives discreetly, still not in uniform. (Obs. 36)

Identifying the medical team’s compliance with arrival and departure times was challenging, as members arrived at different times and had overlapping duties in other hospital areas, such as the outpatient clinic and the emergency department. Health assistants were punctual.

#### 3.3.4 Knowing self

##### 3.3.4.1 Lack of critical team reflection

Moments of reflection on professional practice often occurred when actions do not align with personal expectations. However, this capacity for reflection was not observed within the team.

“*I must organize my shift better; I don’t have time to look at files. How come I didn’t know that cardiology had come to see the patient? I ended up handing over the wrong information*”. (Obs. 10)

(…) the nurse and the health assistant at the entrance to the room, with gown and gloves on. The nurse asks: “*how long is he going to be there? This is clueless*”. She pulls back the curtain and says, “*do you think this is the best time to listen to him? we’re in the middle of the bath, he’s undressed*”. The doctor replies, “*you’re right, I can come back later*” and leaves the unit without saying anything to the patient. (…) The nurse tells the health assistant that she may have been too harsh with the doctor “*I could have said it differently, don’t you think? Sometimes it feels like I don’t have a filter*.” (Obs. 41)

##### 3.3.4.2 Difficulty in managing emotional demands

Healthcare professionals struggled to recognize, understand, and manage the emotional demands of their profession, as well as their ability to handle stress in life-threatening situations.

HP: “*I’m going to be looking after that patient again; it’s getting to me (…) he is getting weaker and weaker, and always worried about his wife…*” (Obs. 15)

The nurse always had a calm attitude and didn’t transmit any anxiety to the patient, the health assistant or the doctor. (Obs. 12)

#### 3.3.5 Clarity of beliefs and values

##### 3.3.5.1 Influence of personal convictions on professional practice

Personal convictions shaped how staff approached their work at an individual level, interacted with older adults, and made decisions. The alignment or misalignment of these beliefs with person-centered care principles affected the way staff delivered care and engaged with patients.

*Yesterday afternoon HP warned against leaving this patient sitting (…) they said it had been difficult to manage the work. (…) HP: “It’s not my patient, but just so you know, I ignore this kind of information. I assess what’s best for the patient, I’m not worried about what my colleagues in the afternoon say”.* (Obs. 15)

In relation to the practice environment dimension, the findings highlighted a collaborative team culture, yet there were significant barriers to shared decision-making across professional groups due to hierarchical structures. The spontaneous and informal discussions observed revealed concerns with vital signs, complications, or therapeutic issues. While valuable, these interactions did not always provide a platform for effective teamwork. The shortage of specialized nurses and the absence of formal multiprofessional meetings constrained collaborative and holistic care approaches. Additionally, the unit’s physical layout, with limited shared spaces, reinforced professional silos. These findings underscore the importance of creating formal spaces for interprofessional communication and enhancing the skill mix to ensure consistent, person-centered care.

#### 3.3.6 Appropriate skill mix

##### 3.3.6.1 Managing patient-to-staff ratio

The staff distribution by shift aimed to balance the different functions and levels of experience, ensuring complete coverage of patient care and the health assistants kept track of the number of nurses per shift. However, staff are occasionally absent from the service for health or family reasons without being replaced, putting the ratio into question. Despite the higher workload, staff showed readiness to take on patients who were not initially assigned to them and adjusted their care planning according to the events arising during the shift.

One of the HP (…) takes over the shift of the colleague coming in at 9 a.m. (…), while another HP advances some activities during the shift handover, bearing in mind that today, the patient ratio has changed, and, therefore, they will have more patients in their care. (Obs. 25)

The night shift HP starts the shift handover and mentions that one of the morning colleagues will not attend (…). They must have more patients assigned, so the work distribution has changed. Everyone tries to find out who the new patients are without commenting or wasting time. (Obs. 25)

##### 3.3.6.2 Balancing specialization and experience in care

There were different levels of professional experience among the staff, and an effort was made to maintain a balance between the teams, with the concern to guarantee the quality of the care provided. Concerning specialization, the gap in the nursing team stands out. Of the 50 nurses on the team, only one specialized in medical-surgical nursing and two in rehabilitation. Several shifts were observed without a rehabilitation nurse. Staff recognized the shortage of rehabilitation nurses and its impact on care provision.

*The nurse manager reminds all nurses to be attentive when handing over shifts to newly arrived colleagues. “It is important not to change team members because it causes instability and insecurity. They should be placed in the teams of those who have accompanied their integration into working life and who know their difficulties.”* (Obs. 25)

*The doctor asked how the patient’s tracheostomy closure training had been going. The nurse said there had not been much progress because there was only one rehabilitation nurse, and he did not come in on weekends.* (Obs. 31)

#### 3.3.7 Shared decision-making systems

##### 3.3.7.1 Lack of multiprofessional meetings

There was no formal time or place for healthcare professionals to meet with other professional groups. Information sharing occurred spontaneously in the medical and nursing rooms, bedrooms, or corridors. However, the need to create moments for sharing information and discussion between the various professionals was expressed.

*Are four doctors outside a room discussing treatment options (…) One of the doctors starts sharing information about one of the patients (…) and approaches the nurse in the room to get more information about him.* (Obs. 39)

*“How come we are putting off this referral to palliative care for so long?” (…) The HP said she was disappointed that there was no multidisciplinary meeting to discuss these situations as a team (…). “We end up feeling that we do not have a voice.”* (Obs. 25)

##### 3.3.7.2 Opportunities for active involvement in decision-making

In team meetings, there were opportunities to actively influence care decisions within the different professional groups through case discussion. However, challenges arose in find mutual solutions among professional groups, despite efforts to collaborate.

During the shift handover, the HP mentioned that one of the patients needed a central venous catheter to continue administering the therapy that had been prescribed (…). HP: “Although this has already been communicated to the physicians’ team, they don’t seem alert or willing to insert the catheter.” (Obs. 25)

After talking with the patient, the HP (…) discussed as a team what the treatment priorities would be, considering the motor deficit, the presence of easy fatigue and the need for respiratory rehabilitation to achieve the independence that was desired. They have thought of treatment options that they will consider with the family. (Obs. 9)

#### 3.3.8 Effective staff relationships

##### 3.3.8.1 Collaborative teamwork

Team members demonstrated adaptability, effective task management, and a commitment to collaboration toward common goals.

The doctor approaches the nursing room (…) and says that a patient needs his central catheter “rotated”. The nurse asks if she should prepare the material immediately and stops the computer records, she was making. She goes to the workroom, gathers the material, asks the health assistant to organize the space in the unit for the procedure. (Obs. 45)

##### 3.3.8.2 Mutual support among staff

Team members communicated and supported each other respectfully and professionally, reflecting shared values of mutual trust, support, and respect in everyday situations and during clinical instability. The role of leadership in facilitating teamwork during challenging moments was also observed.

During the shift, a HP had a personal problem (…) and ended up leaving the ward. The colleague who took over his tasks showed her support and reassured her, “Don’t worry.” (Obs. 8)

The head nurse becomes aware of the inpatient decompensation (…) and comes to the room to check if she needs help. (Obs. 12)

#### 3.3.9 Power sharing

##### 3.3.9.1 Equity among professionals in the same group

A collaborative approach to resolving differing opinions was identified among professionals in the same group, who recognized that leveraging specialized knowledge could address uncertainties and enhanced the quality of care.

Two physicians have different opinions. They all decide to call the gastroenterology specialist to explain their case and ask for advice. (Obs. 39)

##### 3.3.9.2 Impaired power sharing between professional groups

In contrast, misalignments between different professional groups represented a missed opportunity for interprofessional dialog.

The doctor (…) enters the room and says, “*I have seen the patient (…). I do not know what has happened but try removing the NIV and see if she can tolerate it*”. The nurse (…) says she will remove the NIV without asking the doctor any questions or providing any more information, even though she knew that when she changed the mask in the morning, the patient had low oxygen saturation. (Obs. 21)

#### 3.3.10 The physical environment

##### 3.3.10.1 Unit architecture on the care environment

The unit’s architecture consisted of a long corridor with nine patient rooms on one side and offices for nurses and physicians on the other. The walls were painted light yellow with informational posters for patients and visitors. All rooms had windows with natural light and views of the adjacent building and were equipped with televisions. No remote control was available for patients. The offices for nurses and physicians had windows facing the city, round meeting tables in the center of the room, and several computers were distributed on desks along the walls.

##### 3.3.10.2 Balancing ward dynamics with patient care

Especially on weekdays, the ward environment experienced abrupt transitions, with unforeseen tasks arising simultaneously and professionals adjusting to the change of pace (e.g., discharges and admissions, complementary diagnostic tests, and patient physiological decompensation). During morning shifts, there was hustle and bustle in the corridor, which was full of equipment, and people moved around (blood analysts, physiotherapists, material delivery, food distribution, cleaning staff). During the shift handover, the silence was only interrupted by patient bells. Most of the day, older adults were sitting in armchairs or lying in bed. Still, during the visiting period, there was bustle again, with people coming and going and interacting with patients and staff.

##### 3.3.10.3 Challenges related to patient room design

Almost all care activities took place in the patient unit. Each room could accommodate up to three patients, with a curtain in between for privacy, and a bathroom and shower inside. Although inside the room the atmosphere was calm, high noise levels were observed in the corridor, and the room’s door was always open.

“*The equipment has squeaky wheels*” (Obs. 31), “*people spoke loudly (…) shouting in the corridor to their colleague at the other end of the service*” (Obs. 32), and “*the phone rang and rang without being answered*.” (Obs. 31)

#### 3.3.11 Supportive organization systems

##### 3.3.11.1 Promoting staff involvement in practice development

Staff were encouraged to participate actively in working groups, such as practice improvement projects (e.g., prevention of falls and pressure ulcers), but work conditions still needed to be created (e.g., no time assigned in the schedule).

##### 3.3.11.2 Ensuring access to training opportunities

Different training activities were provided for each professional group in the department, with few specifically aimed at health assistants. At the organizational level, training covered common areas such as infection control, life support, and risk management. To increase staff participation, training modules were mostly scheduled online.

##### 3.3.11.3 Leadership that cultivates collaboration

The clinical director and the head nurse led the unit. They coordinated efforts to resolve tensions and ensured a collaborative environment. Both maintained an open line of communication with the team. However, none of the leaders were observed to express their support for the PCP clearly, consistently, or frequently.

#### 3.3.12 Potential for innovation and risk taking

##### 3.3.12.1 Divergent approaches to healthcare delivery

Distinct approaches to healthcare delivery were observed among different professionals. On the one hand, a creative ability to circumvent the rules and adapt care was identified. On the other hand, staff followed established routines and demonstrated a reliance on hierarchical authority, rather than personal judgment. The potential for collaborative problem-solving underscored a possible rigidity in rule enforcement that did not always fully consider the patient’s specific circumstances.

The relative asks for two visits to the family member (…). The HP says that (…) the rule is only one visit per day and returns to the nursing room to share what has happened with her colleague. The colleague replies, “Can’t the patient go outside in her wheelchair? She is even sitting down and only has 2 liters of oxygen.” (Obs. 44)

Why did you restrain her? “He laughs embarrassedly and says,” Because the colleague said so. “And you do things because you’re told to, can’t you decide?” (Obs. 10)

The analysis of Person-centered process dimension revealed a commitment to engaging older adults on an individual level, although care was often standardized to streamline routines, occasionally conflicting with personalized approaches. While staff made efforts to align care with patient values, this alignment was more apparent in basic care needs than in significant care transitions. These findings pointed to the need for strategies that balanced routines with flexibility, ensuring that protocols supported rather than hindered person-centered practices.

#### 3.3.13 Working with the person’s beliefs and values

##### 3.3.13.1 Efforts to know older adults

Efforts were observed to get to know older adults, acknowledge discomfort, encourage them to discuss what is important to them, and act accordingly.

The HP (…) says they will prepare for the shower and ask if the patient prefers to eat first. HP: “you must be hungry, it’s getting late.” She says no, “eating before bathing is harmful.” HP: “Ok. Then we will move on to shower.” (Obs. 18)

At the same time as talking, the HP is preparing material to check the peripheral catheter (…). She sits down next to her and begins the procedure without alerting the person to the technique but always chatting amiably and with interest. She turns her head to the side and closes her eyes. HP: “I was trying to distract you with the conversation, I know you don’t like it when I touch the catheter”. She smiles. “You can rest assured that everything is fine with this one” (Obs. 3)

##### 3.3.13.2 Tension between individualized and standardized care

Staff used established care practices to provide uniform care for older adults with similar conditions. In some cases, care interventions were imposed despite a person’s disagreement.

The nurse said that the patient is recovering, being more communicative than in previous days. (…) He did not want to get out of bed and clung to the mattress. However, I still got him up. HP: “*He tolerated it very well and is now lying down again*.” (Obs. 10)

#### 3.3.14 Sharing decision-making

##### 3.3.14.1 Exchange of information on care decisions

Exchanging relevant information between staff and older adults ensured that well-informed care and treatment decisions were made collaboratively. The balance between respecting the patient’s self-reporting and using external input to guide care decisions highlighted the importance of clear communication and active listening in delivering effective, individualized care.

She also asked if the lady wandered around the house. The patient says no. The HP insists, “Not even with a walker? Your daughter told me that she could walk with a walker or support.” (Obs. 19)

In this context, the focus was more on sharing information when the person or their family asked about their current health state and health plan, rather than collecting information about the person and their health history. The family was used as a resource to get more information about older adults.

The HP says that he is going to check the IV line and administer the antibiotic. OA: “Am I taking more than one antibiotic? Until when?” The HP asks if she feels any pain and administers the medication while clarifying her questions. (Obs. 4)

##### 3.3.14.2 Collaborative decision-making in basic care needs

Older adults were involved in decisions to meet their basic care needs, with care planes adjusted collaboratively to prioritize their comfort, satisfaction, and participation.

OA: “*Today, let’s try to go to the toilet with the walker. Do you feel like it?*” The patient says yes. HP: “*Do you want to eat first?*” She asks what the food is. HP: “*It’s a porridge made of cornstarch*.” The woman shows a look of displeasure. HP: “*You don’t like it? Would you prefer something else? I can try to get some bread*.” (Obs. 5)

##### 3.3.14.3 Routine-driven approaches to care transitions

When it came to decisions on care transitions, such as discharge, unit transfer, or diagnostic exams, the routine of the unit prevailed. The lack of specific information or opportunity for the patient to ask questions reflected a hierarchical and task-centered approach, rather than a collaborative and person-centered one.

The nurse calls the health assistant and asks her to pack up the patient’s things as he is about to be transferred. She contacts the unit (…), arranges the equipment, medication, and documentation, and only then informs the patient. He asked why he was being transferred, now that he was used to being there, and expressed concern about a relative’s visit, which was scheduled for later in the day. The nurse says that it was the doctor’s decision. (Obs. 43)

The HP (…) informs you that they are going to leave the service to carry out a test, she doesn’t specify which one, nor the collaboration required. The patient doesn’t ask any questions. (Obs. 32)

#### 3.3.15 Engaging authentically

##### 3.3.15.1 Understanding the person’s perspective

Some staff members demonstrated that they valued, understood and were flexible to the person’s perspective, acknowledged the role of familial support in improving the patient’s well-being, and adapted care accordingly.

In the afternoon nurses decide to facilitate a patient’s family staying longer during the visit, as it is an extended hospital stay and the patient’s condition has worsened. (Obs. 32)

##### 3.3.15.2 Aligning care with patient’s known wishes

Healthcare professionals’ effort to align with patients’ wishes and expectations while promoting active engagement in their treatment and recovery was observed. Balancing empathy with motivational support, contributed to a more satisfactory care experience with common goals.

The patient was asked (…) if he has the energy to start doing some exercises. She says she keeps in pain. HP: “*We are going to try out some exercises; you tell me how much pain you have. Pain is normal, but we have to know when it increases and becomes difficult to bear. (…) It is important to do these exercises so that you do not lose any more muscle mass (…) and do not lose the mobility you had before*”. OA: “*That is what I wanted most*.” HP: “*So, let’s work for it!*” (Obs. 23)

#### 3.3.16 Being sympathetically present

##### 3.3.16.1 Demonstrating understanding and support

Healthcare professionals demonstrated compassion when addressing older adults’ expressions of sadness or concern. Their responses balanced reassurance, acknowledgment of emotions, and humor to alleviate distress and promote comfort.

The patient (…) says that her husband was visiting her and thought she was more tired than yesterday. HP: “And what do you think?” OA: “I think I’m the same.” HP: “But do you feel worse?” (…) OA: “I went in the shower today; it was my first time.” HP: “And it went well, didn’t it? It’s natural to get tired in the shower the first few times. (…) Your husband wants you to go home” she laughs “If you’ve been in the shower, it’s because you’re better, a few days ago you couldn’t even get out of bed. It’s one step at a time.” (Obs. 13)

The doctor says, “I don’t have good news. I had promised you that today would be your discharge day, and everything was planned for that, but we had the result of a test that we weren’t expecting, so we will have to postpone your discharge. It’s not going to be today anymore.” The lady looked visibly sad. The doctor apologized for having created this expectation (…) and explained that she would have to take medication for a few days. She seemed to understand but kept her sad face. The doctor jokingly said: “It’s not so bad; you’re still here with this lady that you talk to a lot, and you need to be at 100% to get back home; the grandchildren give you a lot of work, don’t they?” (…) The lady ended up breaking her sad face. (Obs. 7)

#### 3.3.17 Working holistically

##### 3.3.17.1 Integrating all dimensions of the person in care

Some healthcare professionals demonstrated their commitment to providing positive care experiences that integrated more than a physiological dimension. Despite resource limitations, they made effort to balance compassion, creativity, and effective communication, fostering the fulfillment of patient’s needs.

HP: “*Do you want to wash your hair? (…) I’ll see if there’s any shampoo*.” Faced with the fact that she had no personal hygiene products, she asked, “*Do you ever receive visitors? Can they bring shampoo from home?*” She replies that she has no shampoo at home because she always goes to the hairdresser to wash. The HP said that she would use the unit’s shampoo, which probably wasn’t that good, but she would do her best to look as good as when she left the hairdresser. (Obs. 2)

##### 3.3.17.2 Recognition of biomedical tasks in practice

The observations suggested that healthcare professionals prioritized task completion over immediate responsiveness to patient bells or visitors. Nurses and health assistants divided tasks, focusing on administering medication, hygiene, and comfort care to ensure efficiency in a high-demand environment. This approach might have led to feelings of neglect or dissatisfaction among patients, and visitors could have perceived staff as unavailable or inflexible.

The nurses split up and carry out the tasks at the end of the shift. One of the nurses takes the medication trolley to one end of the service and starts administering oral and IV medication. The other begins hygiene and comfort care with the health assistant at the opposite end. Bells are rung constantly. The HPs follow the sequence of the rooms and only answer the bells when they get to the room. (Obs. 35)

Visitors ring the doorbell (…). The HP in charge of the patient is busy preparing the therapy and tells the relative to wait outside for her to finish what she is doing. (Obs. 32)

## 4 Discussion

Observations conducted across all weekdays and shift patterns offered a balanced perspective of the unit’s activity, aligning with the literature on capturing authentic healthcare interactions through varied timings (28), frequent, brief interactions typical of high-demand care environments, and consistent with findings by McLaney et al. (29) on staff’s need to adapt to shifting demands. Nurses were central to observed interactions, which mirrors the Portuguese healthcare context, where nursing staff frequently lead inpatient care. This aligns with the Ministry of Health’s data, reflecting nurses as primary caregivers in such settings (30). The observed profile of older adults, who are predominantly female and present with chronic conditions requiring high dependency, is characteristic of internal medicine units that manage complex, long-term care needs in aging populations (31). These demographic and interaction patterns provided the context for interpreting the unit’s workplace culture and person-centered care practices.

The thematic analysis reflected how workplace culture shapes person-centered care in the internal medicine setting, according to the PCPF. The diversity of identified subthemes underscores the complexity of care delivery, reflecting both theoretical concepts and their practical manifestations. Reflective sessions with 15 healthcare professionals supported the validation of findings, adding depth to the interpretation.

The professionally competent theme highlighted the technical knowledge of healthcare professionals, often aligned with biomedical expertise but not always translating into holistic care. This contrasts with McCormack et al. (3) model of competence, which encompasses knowledge, skills, and attitudes for individualized care. While experienced staff effectively anticipate older adults’ needs, routine practices often take precedence over personalized care, suggesting opportunities for reflective practice and mentoring for newer staff (32).

Effective communication, respect and compassion were evident among staff, particularly in interactions with older adults and families, which aligns with the cornerstones of the PCP (3). Collaborative communication within professional groups supported patient care, but the lack of formal interprofessional meetings limited broader information exchange and collective decision-making, indicating the potential benefits of structured communication forums.

The commitment to the job theme revealed dedication to high care standards, although non-compliance issues (e.g., wearing artificial nails) reinforced findings by Brooks et al. (33) on factors affecting healthcare professionals’ compliance. Staff demonstrated strong patient engagement, through intentional involvement with older adults’ and family to understand their needs and preferences despite the workload pressures that sometimes led to task-oriented disengagement once routine care was complete.

In the knowing-self theme, although moments of self-reflection were observed, professionals cited stress, routines, and limited structured reflection time as barriers to regular critical reflection. Formalized reflective practice could address this gap, supporting staff’s personal and professional growth in line with PCPF principles (3). Staff also faced emotional challenges in life-threatening situations, underscoring the need for coping mechanisms to manage the emotional demands of care.

The clarity of beliefs and values theme showed that personal convictions significantly influence care delivery, sometimes aligned but other times diverging from person-centered principles. Cultivating awareness of these values could enhance consistency in person-centered approaches (34).

In the prerequisites construct, the strengths identified include the staff’s strong technical expertise and biomedical knowledge, effectively anticipating patient needs. Communication and compassionate interactions with patients and families align well with PCP principles, and staff commit to delivering high-quality care. Additionally, collaborative relationships and mutual support among team members foster a positive and supportive work culture. Areas where further development is needed guarantee professional standards, deepen knowledge in non-technical areas and enhance support for reflective practices individually and collectively. Emotional challenges in life-threatening situations reveal a need for stronger coping mechanisms and alignment of personal values with PCP principles to ensure consistency. Lastly, high workload pressures, stress, rigid routines, and insufficient time for reflection hinder professional growth and contribute to task-oriented practices, reducing opportunities for meaningful patient engagement.

Regarding the appropriate skill mix theme, the observed staff–patient ratios and skill composition are largely aligned with regulatory standards (30, 35), although unplanned absences disrupted ratios and increased workloads. Ensuring staff levels is essential for sustaining a high level of care and preventing burnout among staff (36, 37). Limited specialization, particularly in rehabilitation nursing (35), highlights a significant gap that targeted recruitment and skill development programs could address.

In the shared decision-making systems theme, the absence of formal multiprofessional meetings and structured interprofessional communication restrict opportunities for information sharing, inclusive decision-making, leading to hierarchical dynamics where physicians often dominated care plans despite nurses’ relevant input (38). The example of a nurse following the physician’s directive without question, despite having pertinent information, suggests a hierarchical culture that may stifle open communication and hinder person-centeredness (39). Encouraging more structured, inclusive decision-making processes would empower all team members to participate actively, promoting a balanced and person-centered workplace culture (40).

In the effective of staff relationships theme, collaborative relationships and mutual support create a harmonious work environment, fostering resilience and cohesion in patient care (41). The observed readiness to assist colleagues and seek advice reflected a supportive culture essential in high-pressure care settings, contributing to the unit’s collective strength.

The power-sharing theme reveals a dichotomy between intra and inter-professional dynamics. Collaborative openness within professional groups and, conversely, a hierarchical culture that may stifle open communication and hinder person-centeredness was observed (39). Addressing these power imbalances is crucial for fostering an environment where all professionals feel valued and empowered to contribute to patient care (40). The physical environment, comprised of long corridors, contributed to isolation between staff and older adults, and noise from high activity levels detracted from patient privacy (42). Organizational adjustments, such as noise reduction and dedicated quiet areas, could improve patient comfort and privacy, enhancing the patient-centeredness of the environment.

The supportive organizational systems theme revealed the importance of practice development and training opportunities in sustaining a person-centered culture, as continuous learning reinforces person-centered principles (42). Although staff were encouraged to participate in quality improvement projects, there was limited emphasis on fostering reflective practice and team dialog. Providing dedicated time for such reflective activities could empower staff, fostering improvements in sustainable care. Expanding training to include diverse professions and specific skills would enhance team competence, supported by leadership that prioritizes individual and team growth (12, 43).

The potential for innovation and risk-taking theme highlighted a tension between adhering to protocols and exercising professional judgment. While some staff demonstrated creativity in adapting care to individual needs, others followed routines without question, underscoring the need to balance innovation with safety to advance the PCP (43). Establishing a culture that encourages both risk-taking and adherence to safe practices is essential for meaningful person-centered care. With respect to the practice environment construct, challenges related to staffing, specialization gaps, and hierarchical structures impacted collaborative care. Although the environment was largely supportive, power imbalances and ward layouts that physically isolated older adults presented obstacles to effective PCP. Additionally, a lack of formal meetings and practice development time constrained continuous improvement. Addressing these gaps, such as by introducing multiprofessional meetings, would promote collaborative decision-making and shared accountability (44).

In working with the person’s beliefs and values theme, staff showed sensitivity to individual preferences, engaging personally with older adults. However, the necessity of standardized care sometimes conflicts with personal approaches, where established protocols could overshadow individual needs. Balancing flexibility with protocol adherence remains a core challenge for PCP (45, 46). The theme of sharing decision-making revealed variability in the involvement of older adults, with decision-making often limited to responses rather than proactive engagement. Encouraging a more collaborative approach could further align care with patient values (39). Older adults should be seen as active facilitators of decision-making (45, 46). Involving families as resources also enhanced PCP, recognizing the older adults’ broader context. However, major care transitions often followed organizational routines, highlighting areas for improvement in shared decision-making practices (39). The behaviors of engaging authentically and being sympathetically present aligned with the PCPF’s holistic care, illustrating staff efforts to connect genuinely with older adults (9). These actions fostered patient trust and a sense of security. However, a duality exists between holistic care and task-oriented approaches, where routine-based methods prioritize clinical efficiency over emotional and psychological needs (17). Strategies to integrate individualized approaches with standardized protocols are essential to reinforce PCP principles (47, 48).

In the person-centered processes construct, task demands frequently conflict with individualized care. High task orientation often undermined holistic approaches essential to the PCP, as staff judged competing priorities. Sharp et al. (49) reported similar tensions in acute settings, where task-oriented workflows diverted focus from direct patient care. Care fragmentation, time constraints, and goal-oriented organizations are reported as challenges in transitioning to PCP and factors delaying this change (12, 50). Aligning daily routines with person-centered principles, by training staff to merge standardized and personalized care could mitigate these conflicts, ensuring that protocols support, rather than constrain PCP (48). Additionally, older adults’ involvement in decision-making was limited to basic care needs, remaining opportunities to expand collaboration to include a broader scope of care decision. The study did not clearly address the willingness of older adults to participate in decision-making. However, previous studies in Portugal have shown a preference among older adults for healthcare-led decision-making in acute care, possibly due to dependence and loss of control in institutional settings (51, 52). Enhancing emotional relationships and personalizing information sharing could foster a collaborative environment, encouraging patient involvement and aligning care with individual values (48).

Variability across subthemes, such as working holistically, suggests inconsistency in how different professionals implement PCP. While some healthcare professionals make efforts to align care with the individual preferences of patients, routine practices often take precedence. This tension between adherence to established guidelines and the flexibility required to address personalized patient needs is a challenge throughout the unit. The inconsistent approach to care leads to older adults experiencing ‘person-centered moments,’ reinforcing the need for a more consistent workplace culture (47, 53).

The prioritization of task demands during shifts revealed a tension between essential aspects of PCP that span several themes across all dimensions of the PCPF, including professional competence, commitment to the job, knowing self, potential for innovation and risk-taking, sharing decision-making, and working holistically. This tension suggests that the task-oriented nature of work may conflict with the holistic, individualized approach that PCP requires. As a result, staff may struggle to integrate these core elements of PCP while meeting the demands of a busy clinical environment. Sharp et al. (49) identified this tension in acute settings, leading to missed aspects of care, especially non-technical ones. To enhance PCP it is crucial to emphasize strategies that support the alignment of daily routines with person-centered care principles (47), such as training staff to integrate standardized care with personalized approaches, ensuring that protocols serve as a foundation rather than a constraint.

The absence of systematic multiprofessional team meetings impacted several themes, including knowing self, shared decision-making systems, power sharing, supportive organization systems, and the potential for innovation and risk-taking. Regular meetings could facilitate professional growth and collaborative decision-making, promoting shared responsibility and comprehensive care planning (44). These structures are essential for creating an environment where each professional’s contributions enhance person-centered outcomes. The hierarchical culture within the unit further complicates this issue. Physicians often dominate care planning, even when nurses have pertinent information that could contribute to decision-making. This power imbalance stifles open communication and limits the active participation of all team members in the care process. Addressing these hierarchical dynamics is crucial for fostering a more collaborative, patient-centered environment.

Professional development and reflective practice are also central issues. There is a significant gap in opportunities for continuous learning and structured reflection within the team. Healthcare professionals could benefit from formalized reflection periods to assess their practice and enhance their understanding of person-centered approaches. The lack of time and support for reflection and critical thinking restricts personal and team growth. Addressing these challenges through professional development, critical reflection, collaborative structures, and enhanced patient engagement practices remains essential for cultivating a sustainable person-centered culture. PCP just can thrive within an established care culture that prioritizes person-centered values and practices (53).

Overall, these transversal subjects underscore the importance of addressing organizational and structural challenges, such as communication improvement, collaborative culture fostering and, reflective practice support, to strengthen the PCP implementation and cultivate a sustainable person-centered culture (53).

As limitations of the study, the data collection was confined to one unit in a secondary public hospital, keen to understand the work culture, so the findings are not generalizable.

We identify potential biases in data collection based on observations and participant narratives, as they might omit critical aspects of interactions that were neither directly observed nor valued by the observer. Additionally, the recent appointment of the head nurse is a significant event that could introduce variations in data, depending on its impact on the work environment.

Owing to the study’s methodological characteristics, the individual and organizational levels of intervention were considered in the interpretation of the findings. A deeper understanding of how the PCP is framed at the structural level and supported by health system policies could enhance the interpretation of the results.

As a strength, exploring person-centered concepts within a theoretical framework grounded in an established theory bolsters the existing knowledge of PCP in the field. Understanding the work culture of the unit based on PCPF offers added value in guiding the transformation of the PCP by defining areas for prioritization. To our knowledge, this is the first study to use an inductive approach based on this theoretical framework to describe the work culture in an acute healthcare setting. The established methodology, capturing behaviors in all shift patterns, including all professional groups and older adults, and validating the interpretation of the results in reflective sessions, lends credibility to the results.

## 5 Conclusion

This study contributes to a broader understanding of how work culture is experienced in an internal medicine unit through the PCPF lens and deepens how theoretical concepts can be identified in practice.

The diversity of subthemes identified reflects the complexity of care delivery and underscores the strengths and challenges of aligning it with person-centered principles. It offers guidance for targeted interventions to foster a person-centered work culture.

Key strengths include the healthcare professionals’ technical competence, commitment to high-quality care, and compassionate communication with patients and families. Supportive staff relationships foster resilience and cohesion, contributing to a positive work environment. These elements align with the core principles of PCC and underscore the opportunities for further growth.

However, several challenges were identified. Task-oriented practices often take precedence over holistic care, and inconsistencies in PCC implementation result in fragmented patient experiences. Hierarchical dynamics constrain collaborative and inclusive care planning. The absence of structured multiprofessional meetings limits effective communication and shared accountability, while the lack of reflective practice opportunities hinders professional growth. Additionally, high workload pressures and routine-driven care reduce opportunities for meaningful patient engagement. Future efforts to address the identified challenges, such as implementing formal multiprofessional meetings, supporting collaborative decision-making, promoting staff engagement in reflective practices, and patient involvement in decision-making, will be crucial in advancing the PCP in this context.

## Data Availability

The original contributions presented in the study are included in the article/Supplementary material, further inquiries can be directed to the corresponding authors.
