# Supplementary material for: Person-centered workplace culture: insights from an inpatient department for older adults with chronic illnesses
Source: Front Med (Lausanne). 2025 Feb 26;12:1532419. doi: 10.3389/fmed.2025.1532419 (PMC11897487; doi:10.3389/fmed.2025.1532419)
Supplement: Supplementary file 1 [file Table_1.docx]

**Protocol for using the Workplace Culture Critical Analysis Tool® (WCCAT)**

| **Phase** | **Description** |
| --- | --- |
| Pre-observation | - Engage with senior staff to identify the inpatient participants. - Assess the participant's inclusion criteria. - Engage with multiprofessional groups to explain and clarify any doubts about how the study will proceed. - Provide written and verbal information. - Distribute consent forms. - Be aware of events (meetings, training sessions, audits). - Reflect on the values and beliefs of the context. - Define the focus of the observation. - Discuss concerns with all the study authors. |
| Observation | - Each observation session is to last at most 1 hour. - WCCAT proforma will act as a prompt for data collection. - The researcher will move around the ward to collect as much data as possible. - The observations will be audio recorded, and field notes (comments, questions) will be taken to support the audio description. |
| Raising awareness | - Meet with staff after observation as soon as possible to clarify any queries. - Provide initial feedback if staff asks for it. |
| Reflective dialogues | - Use field notes and staff feedback to identify issues for discussion and exploration in reflective sessions. - Critical dialogue around findings from observations (compare what has been observed with what they believe happened in practice). |
