# Supplementary material for: Person-centered workplace culture: insights from an inpatient department for older adults with chronic illnesses
Source: Front Med (Lausanne). 2025 Feb 26;12:1532419. doi: 10.3389/fmed.2025.1532419 (PMC11897487; doi:10.3389/fmed.2025.1532419)
Supplement: Supplementary file 2 [file Table_2.docx]

**Reflective Sessions Script**

**Objective**: To share and reflect on the results of the field observation.

**Duration**: 1h00

**Participants**: Healthcare team (physicians, nurses, health assistants, physiotherapist)

**1. Background** (10 minutes)

- Introduce the researcher and thank everyone for coming.
- Explain the session's aim: to share and reflect on the results of observing the unit dynamics, with a focus on person-centered practice.
- Present the session's structure: present the results by construct, followed by discussion. Depending on the number of participants, divide into small reflection groups and then share collectively.

**2. Presentation of the results** (10 minutes)

- Explain the deductive process of obtaining the themes based on the Person-centred Practice Framework.
- Present the results with examples of situations observed that illustrate the concepts attributed to the themes.
- Reinforce the importance of basing the analysis on a person-centered approach.

**3. Reflective group discussion** (35 minutes)

- Divide the team into small multi-professional groups.
- The researcher goes around the various groups to facilitate discussion and reflection on practices.
- Do you agree or disagree with the interpretation of the data? Why? Do you identify with what has been described? (ask for examples of situations that support this opinion).
- Ask each group to share their reflection.

**5. Closing and feedback** (5 minutes)

- Summarize the main points discussed.
- Thank everyone for their active participation.
- Reinforce the importance of person-centered care and the commitment to continuous improvement.
